# Mental association of time and valence revealed with a novel chronometric approach: The positive-future effect

**DOI:** 10.3758/s13421-025-01715-y

**Published:** 2025-05-14

**Authors:** Markus Janczyk, Katharina Tucholski, Barbara Kaup, Rolf Ulrich

**Affiliations:** 1 Department of Psychology, University of Bremen, Hochschulring 18, 28359 Bremen, Germany; 2 Department of Psychology, University of Tübingen, Tübingen, Germany

**Keywords:** Time, Valence, Congruency effects

## Abstract

Two recent studies utilized indirect response procedures (i.e., a sentence completion task and the Implicit Association Test) and suggest that people evaluate the future more positively than the past (Kaup et al., *Frontiers in Psychology, 12,* 612,720, 2021; Ulrich et al., *Memory & Cognition, 52,* 444–458, 2024). This present article reports a novel chronometric approach and a self-report study examining whether this relationship can be observed consistently. In one part of the chronometric study, participants were instructed to respond verbally with the words “past” and “tomorrow” to negatively and positively connotated words. In the positive-future condition, participants responded with “tomorrow” to positive and “yesterday” to negative words; in the positive-past condition, they responded with “yesterday” to positive and “tomorrow” to negative words. In the other part, participants responded verbally with “good” and “bad” to time-related words. In the positive-future condition, they responded with “good” to future-related and “bad” to past-related words; in the positive-past condition, they responded with “good” to past-related and “bad” to future-related words. Response times were shorter in the positive-future than in the positive-past condition, suggesting that participants consistently evaluate the future more positively than the past (i.e., the positive-future effect). This strengthens the view that the positive-future effect is robust and general. Several possible mechanisms of why this effect emerges are discussed. The self-report study, in contrast, indicated no significant difference in individuals' perceptions of the past compared to the future. This may be attributed to a positivity bias in recalling past events, which may mask the differences in how people perceive the past versus the future.

## Introduction

Humans possess a remarkable ability to recall past experiences and envision future events. These recollections and anticipations often carry the emotional significance of positive or negative valence. For instance, one may vividly remember failing a driving test or passing it on the first attempt. Likewise, the prospect of an upcoming exam can evoke positive or negative emotions, depending on the individual's perceived level of preparedness. This cognitive capacity for mental time travel, whether into the past or the future, plays a pivotal role in shaping our assessments of the past and the future (e.g., Beaty et al., 2019).

The literature on how people perceive the past or the future is filled with contrasting views and speculations. For example, scholars have proposed various mechanisms that influence a positive outlook on the future. One prominent explanation is the belief in ongoing technological, scientific, and social progress and expectations of personal events that can induce an (unrealistic) optimistic perspective (e.g., Kahneman, 2011; Mitchell et al., 1997; Pinker, 2018; Weinstein, 1980). Several researchers have even suggested that this optimism is correlated with a reduced neuronal sensitivity to negative future information (e.g., Chowdhury et al., 2014; Sharot et al., 2011). In addition, a review of brain-imaging studies reported that activity in the anterior cingulate cortex correlates with positive future expectations in a belief update task (Erthal et al., 2021). Others, however, have argued that the research on this optimism bias provides an overblown picture of people’s optimism (e.g., Harris & Hahn, 2011; Shah et al., 2016). Nevertheless, from an evolutionary standpoint, optimism could serve adaptive functions by motivating risk-taking and goal pursuit in challenging environments, ultimately enhancing survival and reproduction (e.g., Kaup et al., 2021).

By contrast, on the preceding claims about a positive perception of the future, people may also idealize the past. For example, people’s nostalgic memories can promote well-being (see Layous & Kurtz, 2023; Sedikides et al., 2016) and thus create a positive view of the past. While the affect associated with negative memories tends to fade relatively quickly, the affect of positive memories remains strong, a phenomenon called the Fading Affect Bias (for a review, see Williams et al., 2022). Positive events from our past are remembered more frequently than negative ones (Walker et al., 2003). In addition, future events tend to be perceived as abstract (e.g., Bausenhart et al., 2024; Kaup et al., 2024; Trope & Liberman, 2010) or uncertain, potentially evoking negative emotions (e.g., Carleton, 2016; Vazard, 2022) and thus provoking a pessimistic view about the future in contrast to the past. Finally, the nature of news may distort people’s view of the world and produce “progress phobia” (Pinker, 2018, Chapter 4), which probably overvalues people’s positive memories of the past. Thus, nostalgia, positive memories, and a pessimistic view of the future contribute to a positive perception of the past.

It should be noted, however, that these views and speculations about the perception of the past and future usually do not contrast emotional responses to the future with those to the past, leaving unanswered whether people genuinely perceive the future as better or worse than the past. Another bulk of studies, however, has addressed this and directly contrasted the perceptions about the past and the future.

These studies often utilize rating-scale questionnaires to explore connections between time and valence (e.g., Berntsen & Bohn, 2010; D’Argembeau & Van der Linden, 2004; Newby-Clark & Ross, 2003; Rasmussen & Berntsen, 2013). The results obtained with these self-reports are somewhat mixed, however. In one study, participants rated anticipated events more positively than remembered events (Newby-Clark & Ross, 2003). In addition, in a further study, participants were faster to generate positive future events than negative ones, indicating challenges in imagining adverse future events. Rubin (2014) contested these findings, as participants in his study considered troubling future events more negative than troubling past events. However, the context of recalling these events was restricted to specific categories of events involving traumatic experiences. This could have caused participants to view the future as more troubling than the past (but see Walker et al., 2003).

Two recent studies employed a rather indirect approach to assess the cognitive association between time and valence (Kaup et al., 2021; Ulrich et al., 2024). First, participants in the study of Kaup et al. (2021) performed a sentence completion task. They were given an incomplete sentence and were asked to choose from two possible phrases to complete it. Notably, when the initial fragment depicted a positive event, participants predominantly selected a phrase related to the future (e.g., *The tour through the palace … is next week* or *was last week*?). In contrast, when the initial fragment was negative, they tended to choose a phrase associated with the past (e.g., *The tour through the bunker … is next week* or *was last week*?). This observation indicates a cognitive association between emotional valence and time, possibly influenced by a positive perception of the future.

Second, Ulrich et al. (2024) employed the Implicit Association Test (IAT; Greenwald et al., 1998) to assess the potential cognitive association between time and valence. On each trial, a temporal expression (e.g., *lately*, *soon*) or a positively or negatively connotated expression (e.g., *glorious*, *nasty*) was presented to the participants on a computer screen. Participants were instructed to classify this expression as quickly as possible. In one experimental condition, participants were asked to respond with one hand if the expression referred to the past or had a negative connotation and with the other if the expression referred to the future or had a positive connotation. In a second condition, they were asked to respond with one hand if the expression referred to the past or had a positive connotation and with the other hand if the word referred to the future or had a negative connotation. Faster responses were observed in the former than in the latter condition, suggesting that people view the future positively compared to the past.

The IAT has been criticized for the claim that it can measure an individual’s implicit unconscious process, which cannot be measured with explicit measures such as rating scales (for discussions about IAT measurement, see Buttrick et al., 2020; Kurdi et al., 2021; Schimmack, 2021). However, there is less disagreement about the possibility that the IAT measures pre-existing cognitive associations or semantic similarity, as already suggested by Lasaga and Garner (1983) and argued by others (De Houwer et al., 2005; Kurdi et al., 2021; Schimmack, 2021). Such chronometric approaches are particularly attractive for indexing pre-existing cognitive associations because response speed captures automatic processing (e.g., Hübner et al., 2010; Miller & Schwarz, 2021; Ulrich et al., 2015; White et al., 2011). Thus, response speed measures are likely less prone to biases and expectations than rating scales, enabling a more unbiased assessment of how individuals view the past and future.

The IAT is a classification task in which the stimuli (e.g., good/bad/future/past) in each trial must be classified as belonging to either category A (e.g., good/future) or category B (e.g., bad/past). Consequently, the IAT does not directly investigate the proposed connection between time and valence. The present study expands on a different chronometric approach (Eikmeier et al., 2015; Janczyk et al., 2023) to further explore the cognitive relationship between valence and time. This approach is more direct than the IAT, because a stimulus (e.g., good) must be responded to with a word (e.g., future). Hence, in this task, valence and time function as stimulus and response (or the other way round), directly representing a stimulus–response link between the two dimensions of valence and time. Like the IAT, however, this approach is still indirect or implicit in the sense that it does not measure the relationship between time and valence by directly asking participants about it, such as in self-report measures (De Houwer, 2006). Nevertheless, if this approach leads to the same conclusions as Ulrich et al. (2024) reported, the hypothesis that people tend to perceive the future positively compared to the past, or at least, more positively than the past, is strengthened. This would also demonstrate a methodologically robust phenomenon suitable for future research on the perception of the future and the past. Following this chronometric study, we also report a self-report study where we ask participants about their feelings about the past and the future.

## Chronometric study

The chronometric study presents participants in one experimental part with valence stimuli to which a verbal response is required. In the positive-future condition, they are asked to respond verbally with “yesterday” to negatively connotated words and “tomorrow” to positively connotated words. In the positive-past condition, this stimulus–response assignment is reversed; they are now required to respond with “yesterday” to positively connotated words and with “tomorrow” to negative ones. In another part of the experiment, participants respond verbally with “bad” or “good” to time stimuli, also in a positive-future and positive-past condition. If the future is perceived as more positive than the past, one would generally expect shorter response times (RTs) in the positive-future compared to the positive-past condition in both experimental parts – henceforth referred to as the *positive-future effect*. In addition, we obtained scores on the Optimism–Pessimism Scale (SOP2; Kemper et al., 2012) from our participants as a measure of their optimistic personality. As Kaup et al. (2021) reported a stronger association between time and valence for people with an optimistic attitude toward life, we expect positive correlations between the SOP2 value and the positive-future effect.

### Method

#### Open practices statement

This experiment was preregistered at https://aspredicted.org/2TF_VZP and the data can be found on the Open Science Framework at https://osf.io/kvtfq/. All analyses were performed with the R programing language (v4.4.1; R Core Team, 2024) and the following list of R packages: afex (v1.4–1; Singmann et al., 2024), BayesFactor (v0.9.12–4.7; Morey & Rouder, 2024), emmeans (v1.10.4; Lenth, 2024), ez (v4.4–0; Lawrence, 2016), lme4 (v1.1–35.5; Bates et al., 2015), and schoRsch (v1.10; Pfister & Janczyk, 2016).

#### Participants

Forty people (all students, except for one) from the area of Bremen (Germany) participated for course credit or were paid for participation (mean age = 23.62 years, *SD* = 3.23 years; 15 males, 25 females; all participants were right-handed). Thus, the vast majority of the participants were Germans, though we did not ask for their nationality. However, and more importantly, data were only included and analyzed for German native speakers. All participants were treated according to the principles of the Declaration of Helsinki and its Amendments and signed written informed consent prior to data collection. We used *n* = 40 participants as this would have enough power for the interaction effect in which we were interested if we proceeded from an effect size of about *d* = 0.5 for this interaction effect (which we estimated conservatively from our previous research in Ulrich et al., 2024). This would result in *n* = 34 (α = 0.05, 1—β = 0.8, two-sided; see Langenberg et al., 2023).

#### Stimuli and apparatus

Stimulus presentation and response collection were controlled by a standard PC in a sound-attenuated experimental cabin at the Department of Psychology at the University of Bremen. Responses were given vocally and were one of the four German words *gestern* (English *yesterday*), *morgen* (English *tomorrow*), *schlecht* (English *bad*), and *gut* (English *good*). A voice key was used to detect voice onset and thus to measure RT, and the experimenter (sitting outside the cabin) coded the response to provide immediate (error) feedback.

Stimuli were 15 words or short phrases from each of the four possible categories (time stimuli: past-related, future-related; valence stimuli: negative, positive; see Appendix A for a complete list and an analysis of word statistics). Time stimuli were taken from previous work, such as Eikmeier et al. (2015) or Janczyk et al. (2023), and valence stimuli were taken from Ulrich et al. (2024). All stimuli were presented in white against a black background.

#### Task and procedure

Participants responded vocally to a stimulus as soon as possible with a specific assignment. With time stimuli, the positive-future condition was such that past-related stimuli were assigned to the “bad” response and future-related stimuli to the “good” response; the positive-past condition used the reversed stimulus–response assignment. Similarly, for valence stimuli, the positive-future condition was such that negative stimuli were assigned to the “yesterday” response and positive stimuli to the “tomorrow” response; the positive-past condition used the reversed assignment.

Each trial began with a central fixation cross (250 ms) followed by a blank screen (250 ms). Then, the stimulus phrase appeared in the center of the screen and remained on-screen until a response was registered or until 2,000 ms elapsed without registering a response.^1^ The experimenter coded the response (i.e., pressed a key for “gestern”, “morgen”, “schlecht”, and “gut”, plus one for an error response in case of unclear utterances). Errors were visually fed back for 1,000 ms. The next trial started after a blank intertrial interval of 1,000 ms.

All participants performed four blocks resulting from crossing *stimulus type* (time vs. valence stimuli) and the *stimulus–response assignment* (positive-future condition vs. positive-past condition). Each time-stimulus block began with four repetitions of three past-related and three future-related practice words and phrases (i.e., 24 practice trials that were not included in data analyses), which were followed (after a self-paced break) by two repetitions of the 15 past-related and 15 future-related experimental phrases (60 experimental trials). Valence-stimulus blocks were constructed analogously, only negative or positive words and phrases were presented.

The instructions stating the now-required stimulus–response assignment were given on-screen before each block. They emphasized speed while maintaining errors at a low level. Participants were allowed to take a short break after each block.

Half of the participants began with the time-stimuli blocks, the other half with the valence-stimuli blocks. Within the halves, the orders of positive-future and positive-past blocks were counterbalanced across participants, resulting in eight different orders of conditions that were randomly assigned to the participants.

#### Design and analyses

Trials with RTs shorter than 200 ms^2^ or with unclear utterances were excluded from all analyses. For RT analyses, only correct trials were considered. RTs and errors were analyzed with (generalized) linear mixed-effects models with stimulus type (time vs. valence) and stimulus–response assignment (positive-future vs. positive-past) as fixed factors and participants and items as random factors. These analyses were done using the mixed() function of the afex package (Singmann et al., 2024), based on the lmer() and glmer() functions of the lme4 package (Bates et al., 2015). For comparability, likelihood ratio tests (LRTs) were run in both cases to assess the significance of the fixed effects. Holm-adjusted contrasts were run to assess the effect of congruency for both stimulus types separately using the emmeans package (Lenth, 2024). Given that there is – to the best of our knowledge – no undisputed way to calculate standardized effect sizes for mixed-effects models as in the present case, only unstandardized coefficients *b* are reported. However, additional F1, F2, and minF’ analyses of variance (ANOVAs) alongside conventional measures of effect sizes and Bayes factors (BF) for analyses on RTs and percentages errors (PE) are reported in Appendix B.^3^

### Results and discussion

**Fig. 1:**
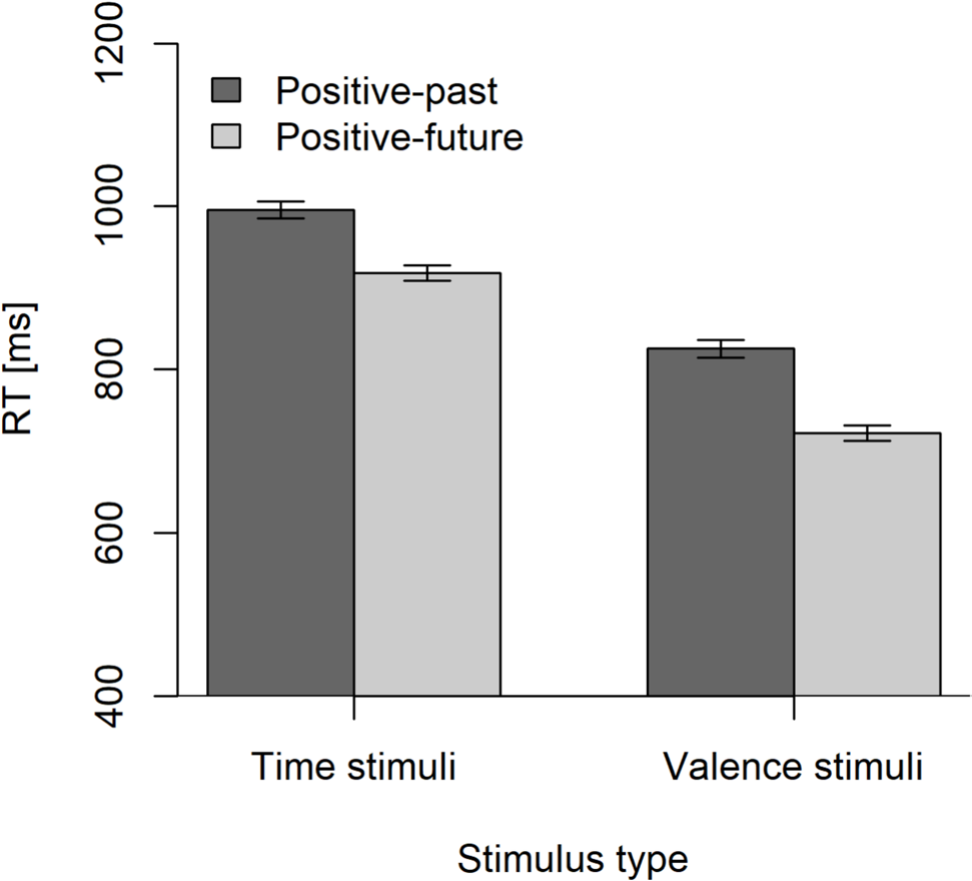
Mean correct response times (RTs) in milliseconds (ms) as a function of stimulus type (time stimuli vs. valence stimuli) and stimulus–response assignment (positive-past condition vs. positive-future condition). Error bars are within-subject standard errors according to Morey (2008)

Mean RTs are visualized in Fig. 1 and summarized with the respective PE in Table 1. Mean RTs were longer with time stimuli than with valence stimuli, and theoretically, most important, for both types of stimuli, a clear positive-future effect is evident. This effect was larger for valence than for time stimuli (104 vs. 78 ms). In addition, more errors were committed in the positive-past assignment compared to the positive-future one, and as for RTs, this effect was larger for valence than for time stimuli (4.25% points vs. 1.74% points).

**Table 1:** Mean correct response times in milliseconds/percentage of errors as a function of stimulus type (time stimuli vs. valence stimuli) and stimulus–response assignment (positive-past vs. positive-future). The last column presents the resulting positive-future effect as the difference between the two assignments

	Stimulus–response assignment		
Stimulus type	Positive-past condition	Positive-future condition	Positive-future effect
Time stimuli	996/5.65	918/3.91	78/1.74
Valence stimuli	826/5.80	722/1.55	104/4.25

A linear mixed-effect model of the full random effect structure (see Barr et al., 2013)

$$\begin{aligned}\text{log}\left(\text{RT}\right)\sim& \text{assignment}*\text{stimulus}\\&+\left(1+\text{assignment}*\text{stimulus}|\text{subject}\right)\\&+(1+\text{assignment}|\text{item})\end{aligned}$$

was used to assess RTs. This analysis revealed all three effects significant, stimulus type: χ^2^(1) = 71.19, *p* < 0.001, |*b*|= 0.19; stimulus–response assignment: χ^2^(1) = 43.96, *p* < 0.001, |*b*|= 0.08; interaction: χ^2^(1) = 5.13, *p* = 0.023, |*b*|= 0.05. Most importantly, the positive-future effect was significant for time stimuli, *z* = 5.77, *p* < 0.001, and for valence stimuli, *z* = 7.30, *p* < 0.001.

The random-effect structure of the generalized linear mixed-effect model to assess errors needed to be reduced because of non-convergence and singular fit warnings, and the final random-effect structure was:

$$\begin{aligned}\text{error }\sim &\text{ assignment}*\text{stimulus}\\ &+\left(1+\text{stimulus}|\text{subject}\right)\\ &+\left(1+\text{assignment}|\text{item}\right).\end{aligned}$$

The main effect of stimulus–response assignment was significant, χ^2^(1) = 49.42, *p* < 0.001, |*b*|= 0.80, as was the interaction, χ^2^(1) = 5.40, *p* = 0.020, |*b*|= 0.74. The main effect of stimulus type was not significant, χ^2^(1) = 0.88, *p* = 0.347, |*b*|= 0.15. The stimulus–response assignment effect was significant for time stimuli, *z* = 3.68, *p* < 0.001, and for valence stimuli, *z* = 6.89, *p* < 0.001. Note that the qualitative pattern of results was the same when using the full random effect structure despite warnings.

The current RT results align with those reported by Ulrich et al. (2024), where participants responded manually in an IAT setting instead of vocally as in the present study. This consistency suggests that the positive-future effect is robust, as it withstands substantial experimental variations and is also evident in sentence completion tasks (Kaup et al., 2021). The estimated effect size (*d* > 1.0, Appendix B) was again large.

#### Correlations with SOP2

The correlation between positive-future effects across the participants was small and non-significant, *r*(40) = 0.21, *p* = 0.194.^4^ Contrary to our expectations, the correlations of the SOP2 score with these effects were even smaller, valence stimuli: *r*(40) = 0.09, *p* = 0.602; time stimuli: *r*(40) = 0.03, *p* = 0.866.

## Self-report study

This questionnaire study explores how participants perceive the past and the future according to their self-report. The goal was to determine whether self-report measures would replicate the data patterns observed in the above chronometric study using the RT method.

### Method

#### Participants

A self-report questionnaire was completed by 56 seminar participants from the University of Bremen's Department of Psychology (mean age = 24.2 years, *SD* = 5.5 years; 10 males, 45 females, one non-binary; 53 right-handed, two left-handed, one ambidextrous).

#### Questionnaire

The questionnaire included two items. The first addressed the past and asked,"How do you feel about the past?"Respondents rated their feelings on a 7-point scale ranging from"negative (1)"to"positive (7)."The second addressed the future and was phrased similarly. The order of the questions was counterbalanced across participants to control for potential order effects.

### Results and discussion

The results are presented with all participants included in the analyses and with only the right-handed participants considered in brackets.

The mean rating for the past item was 4.89 (4.83) and it was 4.96 (4.94) for the future item. A paired-sample* t*-test revealed no significant difference between these ratings, *t*(55) = 0.36, *p* = 0.719, *d* = 0.05 (*t*(52) = 0.55, *p* = 0.586, *d* = 0.08). However, both ratings were significantly greater than the neutral midpoint of 4. A *t*-test for an assumed population mean under H_0_ of 4 revealed a significant effect for the past item, *t*(55) = 5.13,* p* < 0.001, *d* = 0.69 (*t*(52) = 4.61,* p* < 0.001, *d* = 0.63), and also for the future item, *t*(55) = 6.40, *p* < 0.001, *d* = 0.86 (*t*(52) = 5.97, *p* < 0.001, *d* = 0.82), indicating an overall positive tendency. This suggests that, when participants rate their feelings about the past and future separately, they do not perceive the future as more favorable.

## General discussion

The present article reports two studies, a chronometric RT study and a self-report questionnaire study. The chronometric study provides new evidence that the domains of time and valence are cognitively associated. While both domains are linked to the spatial domain (de la Vega et al., 2012; von Sobbe et al., 2019), two recent studies provided the first evidence for a rather direct association (Kaup et al., 2021; Ulrich et al., 2024). Thus, we adopted a new RT method to probe cognitive associations (Eikmeier et al., 2015; Janczyk et al., 2023): In separate blocks of our study, either time responses to valence stimuli or valence responses to time stimuli were required. Large positive-future effects emerged for both the time and the valence stimuli (mapped to the valence and the time responses, respectively). In addition, RTs to time stimuli were longer than RTs to valence stimuli. While we had no hypotheses on this latter outcome, it replicates the results reported by Ulrich et al. (2024). This main effect cannot be attributed to word length or frequency differences between time and valence words (see Appendix A), and hence suggests a processing benefit of valence stimuli.

Descriptively, the positive-future effect was larger for valence stimuli than for time stimuli for RTs and PEs, and the respective mixed-effects models revealed significant interactions for both RTs and errors.^5^ Hence, this effect seems larger with valence stimuli than with time stimuli. Such an asymmetric effect in this direction, however, is in line with results provided by Kaup et al. (2021), where larger choice differences were obtained when the initial sentence fragment contained valence-based information (requiring a choice between future or past sentence completion) as opposed to when it contained time-based information (requiring a choice between sentence completion with a positive or negative valence).

In contrast to the results reported by Kaup et al. (2021), we did not observe a significant modulation of the positive-future effects by the SOP2 score. Rather, the bivariate correlations of this score and the individual effects were negligible. While this outcome does not align with our predictions, it seems worthwhile to consider the main difference between the present study and the one by Kaup et al. (2021). The latter investigated choices, and it seems reasonable to assume that more optimistic people tend to select positive events more often. The present study, in contrast, measured RTs in giving a positive or negative (or past- or future-related) response without any obvious choice. The present results could thus be interpreted to suggest that the accessibility of the required responses, once selected, is independent of individual optimism scores. In addition, SOP2 scores and individual RT effects might be too unreliable to produce a reliable intercorrelation (see Miller & Ulrich, 2013).

The self-report study explored directly how participants perceive the past and the future by asking them. Its results revealed no statistically significant difference between these perceptions. Both past and future perceptions tended to be positive. Self-reports evaluating the past or the future in isolation probably blur differences in the perception of the future compared to the past. For example, as mentioned in the *Introduction*, when remembering past events, people may recall more positive events and filter out negative ones (e.g., Walker et al., 2003). This recall bias about past events could offset the perception about the future (i.e., an optimism bias) and thus conceal the difference between the perception of the past and future. In contrast to self-reports, the RT method does not blur these perceptions, as it is hard to conceive how a biased memory recall could affect RT results in such experiments. This indicates that RT studies are more adept than self-reports in uncovering perceptions regarding the past and future.

This view also fits well with Ulrich et al. (2024), who proposed a modified version of the parallel distributed processing model to explain the positive-future effects observed in their study. According to this model, the mental representations of the past and future oppose each other, inhibiting each other's activation. The same principle is applied to positive and negative valence. However, the mental representations of the past and negative valence excite each other, acting as agonists. Similarly, the mental representations of the future and positive valence are assumed to be agonists. This simple network, comprising four nodes (past, future, negative, positive), generated an activation dynamic consistent with the observed positive-future effects. A further version of this model framework examined whether these effects were merely driven by future time and positive valence since positive valence and future time promote an optimistic view of life (Kaup et al., 2021). To examine this idea, the authors eliminated the exciting links between the nodes of past and negative. However, they retained the exciting ones between the nodes of future and positive. According to this version, the congruency effect for past and negative was predicted to disappear while the one for future and positive was predicted to remain. The observed results were inconsistent with this prediction and thus reinforced the notion that past and negative, as well as future and positive, act as agonists.

Although the model can describe the cognitive processes of how valence and time might activate or inhibit each other, the model is agnostic about why this relationship between time and valence exists. Therefore, the mechanisms that lead to a positive view of the future compared to the past are not captured by this model. The most prominent explanation for this association is that most people are optimistic about the future, as reviewed in the *Introduction* (e.g., Kahneman, 2011; Mitchell et al., 1997; Pinker, 2018; Weinstein, 1980). This optimism is probably a basic mechanism that motivates us to pursue our self-imposed goals even under difficult environmental conditions. In addition, people perceive the future as relatively abstract (e.g., Bausenhart et al., 2024; Trope & Liberman, 2010), which invites wishful thinking about one's future goals (e.g., I will emigrate to the USA as a dishwasher and become a millionaire – The American Dream). One alternative possibility is that the observed association between time and valence merely reflects language experience. Accordingly, people may have learned that words related to the past are often used in negative contexts and words related to the future in positive contexts. However, an analysis based on distributional semantic models excluded this possibility as an account for the observed relationship between time and valence (see Ulrich et al., 2024, Experiment 5). In sum, the robust cognitive association between time and valence most likely reflects a psychological mechanism to perceive the future positively.

The present findings suggest that people generally have a positive outlook on the future. Specifically, our results support the idea that people are more inclined to favor the future without necessarily harboring negative emotions toward the past. This aligns with the common belief that many individuals cherish the past for its nostalgic memories, which contribute to well-being (see Layous & Kurtz, 2023; Sedikides et al., 2016) and a sense of self-continuity (Sedikides et al., 2023). Future studies should further explore people's perceptions of the past and the future.

These considerations also raise the question of how people evaluate the *present* in relation to the past and the future. For instance, Bausenhart et al. (2024) found that people see the future as more abstract than the present (see also Kaup et al., 2024; Trope & Liberman, 2010). However, this distinction was less evident between the past and the present. Consequently, if wishful thinking is the primary reason behind people's positive perception of the future, they may view the future more favorably than the present and the past, while evaluating the past and the present similarly. Not surprisingly, people who imagine themselves as approaching future events also tend to view these events positively (Margolies & Crawford, 2008).

Ulrich et al. (2024) also explored the idea that the connection between time and valence emerges from spatial cognition. In numerous RT studies, participants were asked to determine whether words or sentences referred to the past or future (cf. von Sobbe et al., 2019). It was consistently found that RTs were shorter when participants were instructed to respond with their left hand to indicate the past and their right hand to indicate the future, compared to the opposite hand assignment (e.g., Janczyk & Ulrich, 2019; Santiago et al., 2007; Ulrich & Maienborn, 2010). This phenomenon is known as the *space–time congruency effect*. It is commonly attributed to a mental timeline that runs from left to right in Western cultures (see Eikmeier et al., 2016). Additionally, other studies have shown a mental link between spatial orientation and emotional value. For instance, Casasanto (2009) found that right-handed individuals tended to place positive items on the right and negative items on the left, while left-handed individuals showed the opposite pattern. Furthermore, de la Vega et al. (2012) demonstrated that right-handed individuals responded faster with their right hand to positive words and their left to negative words, compared to the reverse stimulus–response assignment. Crucially, this effect flipped for left-handed individuals. Regardless of the exact explanation for this link, it seems that spatial orientation plays a significant role in how valence is mentally represented, similar to how it influences our thinking about time. This similarity suggests that emotional value (positive/negative) and temporal orientation (past/future) may be connected through spatial cognition. However, Ulrich et al. (2024) observed that both right- and left-handed individuals displayed the same association between valence and time. This finding contradicts the idea that the positive-future effect is mediated by spatial cognition and indicates the involvement of other mechanisms, such as those discussed in the preceding paragraphs. For example, Ulrich et al. also employed nonspatial responses in their Experiments 2 and 3, as in the present RT study. They rejected a spatial account of this effect, because they observed the same congruency effect for left- and right-handed individuals. Because of this, we cannot see a spatial account for the present RT results as an explanation for the positive-future effect.

In summary, the present article reported a novel RT method in a chronometric study and a self-report questionnaire study to examine the potential cognitive linkage between time and valence. Since RT captures automatic processing, the present results of the chronometric study most likely reflect a pre-existing cognitive link: people perceive the future more positively than the past, although this was interestingly not observed in the self-report study. However, the findings from the chronometric study align with those observed by Ulrich et al. (2024), reinforcing the idea of a methodologically robust phenomenon that is promising for future research on the perception of time and valence. For instance, this methodology may be useful for assessing depressogenic beliefs about the future in clinical groups (see Beck & Bredemeier, 2016). Additionally, these methods could be valuable in lifespan studies to explore whether the positive perception of the future changes with age.

## Data Availability

Data are available at https://osf.io/kvtfq/.

## Appendix A: List of stimuli and word statistics

***List of stimuli.*** A list of all stimuli used is provided in Table 2.

**Table 2:** List of words used as stimuli in the experiment

	Past words	Future words	Negative words	Positive words
1	damals *at that time*	absehbar *predictable*	fürchterlich *dreadful*	bestmöglich **best possible*
2	ehemals *formerly*	anstehend **pending*	scheußlich *horrid*	hervorragend *excellent*
3	einst *long ago*	*bald* *soon*	verhasst *hated*	genial *ingenious*
4	einstmals *formerly*	bevorstehend *forthcoming*	gemein *mean*	wunderbar *wonderful*
5	früher *previous*	demnächst *soon*	mies *lousy*	prächtig *marvelous*
6	gestern *yesterday*	in Zukunft *in future*	miserabel *lousy*	erstklassig first-class
7	jüngst *recently*	kommend *to come*	schlimm *bad*	einwandfrei *perfect*
8	kürzlich *recently*	künftig *in future*	widerlich *disgusting*	grandios *terrific*
9	letztens *recently*	morgen tomorrow	hässlich *ugly*	traumhaft *amazing*
10	neulich *recently*	nachher *afterwards*	abscheulich *disgusting*	großartig *great*
11	seinerzeit *back then*	nächstens *shortly*	schrecklich *terrible*	paradiesisch *heavenly*
12	unlängst *recently*	nahend **approaching*	garstig *nasty*	himmlisch *heavenly*
13	vergangen *past*	später *later*	grässlich dreadful	brilliant **brilliantly*
14	vorgestern *the day before yesterday*	übermorgen *the day after* *tomorrow*	böse *evil*	vortrefflich *excellent*
15	vorhin *just now*	zukünftig *in the future*	abstoßend *abhorrent*	herrlich *marvelous*

The English translation is based on the Cambridge Dictionary (online version; assessed 26th of January, 2025), except for those marked with an asterisk (*), which were translated with DeepL

## Word frequency

One potential confound could be that the positive-future effect arises because future and positive words have higher frequencies than past and negative words. This would mean that the word frequency of future words is higher than that of past words, *and* the word frequency of positive words is higher than that of negative words. To assess this possibility, we conducted an analysis of the word statistics for the German terms presented in Table 1, utilizing four databases: (a) CLEARPOND (Marian et al., 2012), (b) SUBTLEX-DE (Brysbaert et al., 2011), (c) Google Books corpus (i.e., Google00pm; see Brysbaert et al., 2011), and (d) the Leipzig Corpora Collection (2018). The latter reports the frequency class of each word rather than a specific frequency count like the other three databases. For each word category in Table 2, we calculated the mean word frequency and the mean frequency class. Notably, some very infrequent words were absent from these databases, including 16 words such as “unlängst” and “garstig” in CLEARPOND, and four infrequent words in SUBTLEX-DE. As Brysbaert et al., (2011, p. 417) recommended, zeros were assigned to these exceedingly infrequent words. Table 3 summarizes the results, indicating that the frequency data do not support the prediction of the aforementioned potential confound. First, the CLEARPOND, SUBTLEX-DE, and Google databases reveal that, while future words are numerically more prevalent than past words, negative words are more frequent than positive ones. Conversely, the Leipzig Corpora Collection shows that past words are more frequent than future words, and positive words slightly exceed the frequency of past words (when looking at Table 3, please note that in this collection a higher class, indicates a less frequent word). To determine significant differences between word categories, we employed the Kruskal–Wallis test, CLEARPOND: χ^2^(3) = 1.63, *p* = 0.652; SUBTLEX: χ^2^(3) = 1.53, *p* = 0.675; Google Books: χ^2^(3) = 12.01, *p* = 0.007; Leipzig Corpora: χ^2^(3) = 10.13, *p* = 0.018. For Google Books, a subsequent post hoc Dunn's test showed a significant difference between the word frequencies of the past and negative categories, and between the past and positive categories, adjusted *p*s < 0.025. For the Leipzig Corpora, the same test indicated a significant difference between mean word frequency of the past and negative word category, adjusted* p* = 0.016. In summary, the complete result pattern is inconsistent with the above-mentioned frequency account of the positive-future effect.

**Table 3:** Mean word frequency as a function of word category and database

	Past		Future		Negative		Positive	
	*Mean*	*SE*	*Mean*	*SE*	*Mean*	*SE*	*Mean*	*SE*
CLEARPOND	38.3	15.2	93.0	46.7	27.9	11.8	22.9	13.5
SUBTLEX	40.1	15.8	94.0	47.6	25.8	9.7	18.4	9.5
Google Books	21.4	9.5	43.7	20.5	3.8	1.7	1.8	0.5
Leipzig	11.5	0.7	12.9	1.4	14.7	0.6	14.1	0.6

CLEARPOND, SUBTLEX, and Google Books provide word frequency per million words. The Leipzig Corpora Collection indicates the word class for each word and importantly, the higher the class, the less frequent the word is

SE = standard error of the mean

***Word length and orthographic neighborhood.*** We also analyzed the word length and orthographic neighborhood of the words in Table 2. The database CLEARPOND contains information about the neighborhood (Table 4). The Kruskal–Wallis-Test did not reveal a significant difference in the orthographic neighborhood, χ^2^(3) = 0.97, *p* = 0.810, but in word length, χ^2^(3) = 9.44, *p* = 0.024. The post hoc Dunn test revealed that positive words were significantly longer than past words, adjusted *p* = 0.014.

**Table 4:** Mean word length and number of neighbors as a function of word category

	Past		Future		Negative		Positive	
	*Mean*	*SE*	*Mean*	*SE*	*Mean*	*SE*	*Mean*	*SE*
Word length	7.45	0.40	7.73	0.51	8.27	0.61	9.60	0.47
Neighbors	1.82	0.67	1.78	0.72	2.23	0.82	0.91	0.16

Neighborhood data were available in CLEARPOND for 13 negative, 11 positive, 11 past, and 9 future words listed in Table 2

SE = standard error of the mean

## Appendix B: Analyses of variance results

Response times (RTs) and percentage errors (PEs) were submitted to stimulus type (time vs. valence) × stimulus–response assignment (positive-future vs. positive past) analyses of variance (ANOVAs) as by-subjects (F1) and by-items (F2) analyses. Based on these results, minimum F'(minF’) was computed following the suggestion of Clark (1973, Eqs. 15 and 16 in his article). For the F1 ANOVA, both factors were treated as repeated measures, whereas for the F2 ANOVA, stimulus type was a between measurement. Additionally, Bayes factors (BF10) were calculated for the F1 ANOVA using the anovaBF() function with standard settings (i.e., rscaleFixed = “medium” for the prior scale for standardized, reduced fixed effects) and 1,000,000 iterations.

For RTs, the F1 ANOVA revealed significant main effects of stimulus type, *F*(1, 39) = 244.29, *p* < 0.001, η_p_^2^ = 0.86, BF = 6.94 × 10^23^ ± 0.22%, and of stimulus–response assignment, *F*(1, 39) = 83.29, *p* < 0.001, η_p_^2^ = 0.68, BF = 1981.69 ± 0.58%, but the interaction was not significant, *F*(1, 39) = 2.67, *p* = 0.110, η_p_^2^ = 0.06, BF = 0.48 ± 1.17%. In the F2 ANOVA, all three effects were significant, stimulus type: *F*(1, 58) = 123.77, *p* < 0.001, η_p_^2^ = 0.68; stimulus–response assignment: *F*(1, 58) = 307.20, *p* < 0.001, η_p_^2^ = 0.84; interaction: *F*(1, 58) = 6.85, *p* = 0.011, η_p_^2^ = 0.11. From these results, minF’ was calculated for each effect. This analysis revealed significant main effects of stimulus type, minF'(1, 95) = 82.15, *p* < 0.001, and congruency, minF’(1, 60) = 65.52, *p* < 0.001, while the interaction was not significant, minF'(1, 68) = 1.92, *p* = 0.170.

The 104-ms positive-future effect with valence stimuli was significant, *t*(39) = 7.77, *p* < 0.001, *d* = 1.23, as was the 78-ms positive-future effect with time stimuli, *t*(39) = 6.37, *p* < 0.001, *d* = 1.01.

For PEs, the F1 ANOVA revealed all three effects significant, stimulus type: *F*(1, 39) = 6.04, *p* = 0.019, η_p_^2^ = 0.13, BF = 1.30 ± 0.99%; stimulus–response assignment: F(1, 39) = 54.26, *p* < 0.001, η_p_^2^ = 0.58, BF = 3,494,598 ± 0.52%; interaction: *F*(1, 39) = 6.25, *p* = 0.017, η_p_^2^ = 0.14, BF = 6.60 ± 0.85%. The F2 ANOVA revealed a significant main effect of stimulus–response assignment, *F*(1, 58) = 39.41, *p* < 0.001, η_p_^2^ = 0.40, and a significant interaction, *F*(1, 58) = 6.57, *p* = 0.013, η_p_^2^ = 0.10. The main effect of stimulus type was not significant, *F*(1, 58) = 1.50, *p* = 0.226, η_p_^2^ = 0.03. Resorting to minF’, the main effect of stimulus–response assignment was significant, minF'(1, 97) = 22.83, *p* < 0.001. The interaction missed conventional significance, minF'(1, 92) = 3.20, *p* = 0.077, and the main effect of stimulus type was not significant, minF'(1, 83) = 1.20, *p* = 0.276.

The positive-future effect of 4.25 percent points with valence stimuli was significant, *t*(39) = 6.03, *p* < 0.001, *d* = 0.95, as was the positive-future effect of 1.74 percent point with time stimuli, *t*(39) = 2.99, *p* = 0.005, *d* = 0.47.

## Footnotes

1 This is a slight deviation from the pre-registration, where we stated that RTs longer than 4,000 ms would be excluded, implying that these were possible.

2 This was not stated in the preregistration. Re-running all analyses with all RTs > 0 ms yielded similar results, however.

3 In the pre-registration, we stated that the mixed-effects models were to be run “additionally,” suggesting that the main analyses focus on the ANOVA results. We present the mixed-effects models in the main text, though, as they appear to be the more appropriate analyses method for the current data.

4 It should be noted that correlations of effects are typically notoriously low due to sole statistical reasons even when the underlying true values are strongly associated (see Miller & Ulrich, 2013).

5 Note that minF’ was not significant and the BF pointed toward the null hypothesis of no interaction for RTs. For PEs, although minF’ just missed significance, the respective BF pointed toward the alternative hypothesis (see Appendix B for more details).
